# P037: Nosocomial influenza prevention using multi-modal intervention strategies; 20-years of experience

**DOI:** 10.1186/2047-2994-2-S1-P37

**Published:** 2013-06-20

**Authors:** A Iten, C Bonfillon, T Bouvard, C-A Siegrist, D Pittet

**Affiliations:** 1 Infection Control Program, University of Geneva Hospitals, Geneva, Switzerland; 2 Department of Health Employees, University of Geneva Hospitals, Geneva, Switzerland; 3 Department of Child and Adolescent, University of Geneva Hospitals, Geneva, Switzerland

## Introduction

Healthcare-acquired influenza is associated with patient morbidity and mortality. Annual vaccination of healthcare workers (HCW) is an important preventive measure to avoid the spread of influenza virus, but HCW vaccination is not mandatory in Switzerland for legal reasons.

Before 1994, HCWs’ influenza vaccination rate at HUG was very low (<10%). The use of various intervention strategies (multiple teaching sessions, institution-wide information campaigns, facilitated access to vaccines) increased HCW vaccination coverage from 14% in 1994 to 21-27% between 1996 and 2008.

Since 2009, an additional measure has been introduced and a badge with the message "I am vaccinated against influenza to protect you" is now given to all vaccinated HCWs. Non-vaccinated HCWs are required to wear a mask during the entire seasonal influenza epidemic period. Apart from 2009 (pandemic A/H1N1 2009), vaccination coverage peaked at 29%. In 2011, only 55.4% of HCWs respected the recommendations (vaccination or correct mask wearing in the proximity of the patients).

## Methods

During the seasonal influenza epidemic 2012/13, all HCWs were obliged to wear a badge that explained their choice to patients/visitors. If vaccinated, this was clearly stated by the badge message; if not, the HCW was obliged to wear a mask during the seasonal epidemic (approximately 12 weeks) and to display a badge with the message "I wear a mask to protect you".

## Results

HCW vaccination coverage reached 37% in 2012/13. In early 2013, an audit quantified the observance of recommendations against seasonal influenza on the hospital wards as follows: 40.4% of HCWs were vaccinated and 30.6% wore the masks correctly, corresponding to 71% of HCWs at HUG who contributed actively to prevent the transmission of influenza virus.

## Conclusion

Influenza prevention requires a multi-modal approach. In countries where HCWs have no obligation to be vaccinated, the choice between the vaccine and mask wear may be a solution to improve the observance of recommendations that aim to prevent nosocomial transmission.

## Disclosure of interest

None declared

